# Exploring a possible association between the occurrence of the *SERPINE1*-675 4G/5G (rs1799889) polymorphism and the increased risk of esophageal cancer in the Caucasian population

**DOI:** 10.1016/j.bbrep.2021.101147

**Published:** 2021-10-05

**Authors:** Anna Agnieszka Klimczak-Bitner, Jan Bitner, Komei Hiruta, Janusz Szemraj

**Affiliations:** a Department of Biomedical Chemistry, Medical University of Lodz, 92-215, Lodz, Poland; b Department of Medicinal Biochemistry, Medical University of Lodz, 92-215, Lodz, Poland; c Graduate School of Science and Technology, Keio University, Japan

**Keywords:** Biomarker, *MMP9* T1702A, Oesophgeal cancer, Polymorphism, rs1799889, *SERPINE1* -675 4G/5G

## Abstract

The goal of this research was to analyze the *SERPINE1* -675 4G/5G (rs1799889) and *MMP9* T-1702A (rs2297864) polymorphisms in esophageal cancer among polish patients, classified as part of the Caucasian population. The analysis of polymorphic gene variants was performed on 35 randomly selected samples excised from patients with esophageal cancer. The tissue specimens were stored as Formalin-Fixed, Paraffin-Embedded (FFPE) blocks. All patients in the sample group were of Caucasian ethnicity. The genotype distribution of *MMP9* T-1702A and *SERPINE1* -675 polymorphisms was analyzed using the Restriction Fragment Length Polymorphism (RFLP) method. A correlation between the expression of −675 polymorphic form of *SERPINE1* and alcohol abuse has been found. Additionally, a correlation between the −675 polymorphism and the subtype of EC developed by the patient has been shown. To the best of the authors’ knowledge, this is the first report investigating the *SERPINE1* -675 4G/5G (rs1799889) polymorphism as a potential candidate for a prognostic biomarker of esophageal cancer.

## Highlights

• FFPE cancer samples serve as good material for detecting *SERPINE1* -675 4G/5G (rs1799889) and MMP9 T-1702A (rs2297864) polymorphisms.
• Expression of −675 polymorphism of *SERPINE1* may correlate with pathoclinical factors for esophageal cancer in Caucasians.
• *SERPINE1* -675 4G/5G (rs1799889) polymorphism may potentially serve as a prognostic biomarker for esophageal cancer.

## Graphical abstract

**Figure:**
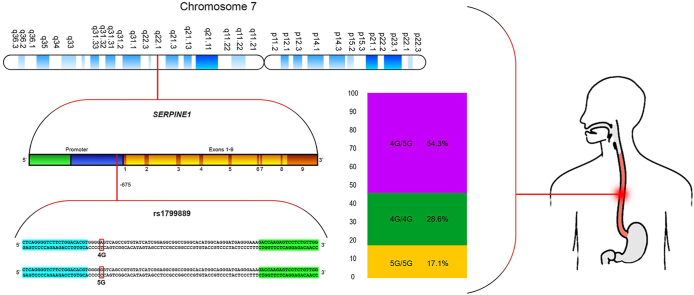

## 1 Introduction

In 2020, a total number of 604 100 new cases of EC were diagnosed worldwide. In the same year, EC was classified along other cancer types and determined the sixth most common cause of death among males (374 313) and the seventh most often incidence cancer type (418 350). Among females, it places ninth in terms of mortality (169 763). In developed countries, it makes for about 6% of all gastrointestinal malignant cancers, while its mortality rate remains very high. It is estimated that among all cancer deaths, EC is responsible for 1 in 20 [1].

Smoking is the primary risk factor for Esophageal Cancer. Compared to lifelong non-smokers, the probability of developing EC among smokers is four to five times higher [2]. Quitting smoking considerably decreases the risk of EC [3]. Alcohol abuse is also a significant risk factor for EC [4]. There is no clear correlation between alcohol consumption and increase in probability of developing EC. There is, however, a correlation between simultaneous smoking and drinking and increased EC risk [5]. In Western Europe and North America, over 90% of all cases of EC are associated with both smoking and drinking. In the case of Asia and South America, the aforementioned correlation remains high, while being lower in more developed countries [6].

Dietary factors also play an important role in EC etiopathogenesis [7]. A poor diet, lacking in fresh fruit, vegetables, dairy, fresh or frozen meat (including fish) and green tea, as well as frequent consumption of fried meat could all potentially increase the risk of EC. Additionally, taking extra doses of microelements, retinol, riboflavin, selenium, vitamin E or zinc does not decrease the risk of EC [6]. In some parts of China, a correlation between consumption of pickled vegetables and the increased number of EC cases has been discovered. Carcinogens associated with development of EC include mycotoxins (including fumonisin B1) and N-nitroso compounds [8]. It is believed that consumption of bracken (*Pteridium aquilinum*) is a factor that increases EC rates in Japan [9].

In Poland, EC makes for less than 2% of all malignant cancer cases. Elderly people are a high-risk group for EC, while EAC is more prevalent among younger patients [10]. Esophageal Cancer is associated with high mortality. Less than 10% of patients diagnosed with EC will survive longer than five years and over 80% of patients die within a year after diagnosis. In the year 2020, 1835 new cases were recorded, which constitutes 0.90% of all new cancer cases in the country. 1736 esophageal cancer deaths were registered in the same year, making up 1,5% of all cancer deaths [10,11].

The gene for plasminogen activator inhibitor-1 (*SERPINE1*) is located on chromosome 7, the molecule itself being a 52 kDa glycoprotein belonging to the serpine super-family [12]. It serves a regulatory purpose in fibrinolysis (breaking down of blood clots), being a principal inhibitor of plasminogen activators both in tissues and urine [13]. It is also proven to regulate cell adhesion, detachment and migration, making its expression an important factor in cancer metastasis [14].

The expression levels of *SERPINE1* appear to be influenced by the polymorphisms in the *SERPINE1* gene [15]. The most frequently studied polymorphism of the gene is located at 675 base pairs (bp) upstream from the start of the promoter, consisting of a single nucleotide insertion/deletion (4G/5G) [16]. It is associated with *SERPINE1* transcription, being possibly involved in its regulation. According to the Centers for Disease Control and Prevention, the split between the 4G and 5G alleles can range from 26.7/73.3% to 52.5/47.5% in various populations [17].

Apart from its association with cancer risk, homozygosity of the 4G allele has been associated with deep vein thrombosis, myocardial infarction and increased risk of miscarriage in pregnant women [18]. The goal of this research was to analyze the *SERPINE1* -675 (rs1799889) and *MMP9* T-1702A (rs3918241) polymorphisms in Esophageal Cancer. It has allowed us to refer back to our previous research, conducted on mRNA level. The 4G/4G polymorphism variant has been associated with increased risk of cancers, colorectal cancer and endometrial cancer especially [17]. Polymorphism analysis allows for DNA analysis of the samples. Previously, the authors have observed a correlation between *MMP9*, *SERPINE1* and miRNA-134 expression and different clinical factors [19]. A correlation between *SERPINE1* genes expression and miR134 has been reported for the first time in the literature [19].

## 2 Materials and methods

### 2.1 Tissue samples from patients

The cancer tissue samples were acquired from 35 patients during resection and subsequently examined. Fragments of non-cancerous tissue were also extracted from the same patients at least 3 cm away from the neoplastic growth. All patients belonged to the Caucasian population. The samples were collected between 2005 and 2013 at the M. Kopernik Provincial Specialist Hospital in Lodz (Lodz, Poland). Each diagnosis was confirmed by licensed pathologists through microscopic examination. All tissue samples were preserved as Formalin-Fixed, Paraffin-Embedded (FFPE) blocks and kept at 4 °C in dry storage conditions. Written informed consent for use of the tissues in research studies was provided by each of the 35 patients. EC is a rare cancer and therefore the number of samples was limited. The Bioethics Committee for Research on Humans at the Medical University of Lodz (Lodz, Poland) approved this project as one following the Declaration of Helsinki (No. RNN/25/12/KE).

### 2.2 Polymorphisms

35 samples taken from patients with EC were selected for gene polymorphism analysis. To verify the results for cancerous tissues, 10% of the samples were repeated.

### 2.3 DNA isolation

DNA from paraffin blocks was isolated using Promega's Maxwell® 16 FFPE LEV DNA Purification Kit (Madison, Wisconsin, United States), in accordance to the manufacturer's instructions. Scraped off sections of paraffin-embedded tissue 1–10 μm thick were placed inside 1.5 ml Eppendorf tubes. Manufacturer-provided incubation buffer and Proteinase K were added, achieving the concentration of 20 mg/ml. The samples were heated up to 70 °C and incubated overnight. After centrifuging, the supernatant was transferred into the provided reagent cartridge. Subsequently, the cartridge was placed inside the Maxwell® 16 MDx Instrument from Promega. Subsequent steps were performed according to the manufacturer's protocol. The quality and concentration of the isolated material was analyzed using the Microliter UV/Vis Spectrophotometer (Picodrop Ltd., Hinxton, United Kingdom) and unused material was stored at - 20 °C.

### 2.4 RFLP-PCR

In order to analyze the genotype distribution of *MMP9* T1702A and *SERPINE1* -675 polymorphisms, the Restriction Fragment Length Polymorphism (RFLP) method was employed [20,21]. The Taq PCR Core Kit (Qiagen, Hilden, Germany) was used for amplification. The final volume of 20 μl included 20 ng of genomic DNA. Data regarding the reaction conditions for *MMP9* T1702A and *SERPINE1* -675 polymorphisms (primer sequence and their annealing temperature, restriction enzymes and their digestion temperatures) were provided (Table 1).

**Table 1:** Primer sequences, restriction enzymes and PCR conditions used in analysis of *MMP9* T-1702A (rs2297864) and *SERPINE1* -675 (rs1799889) polymorphisms using the RFLP technique.

Polymorphism	Primers sequence (5′ → 3′) F – forward; R – reverse	Annealing	Base pair	Restriction enzyme
***MMP9*** T-1702A rs2297864	F: CAAGGTCACATAGCTGGAA R: CACCACGCTTGGCTAAAT	65.0 °C, 35 cycles	315 bp + 38 bp – wild type homozygote (TT) 315 bp + 232 bp + 38 bp – heterozygote (TA) 232 bp + 83 bp + 38 bp – mutant (AA)	*Bsa*BI *(BseJI)*
***SERPINE1*** −675 rs1799889	F: CACAGAGAGAGTCTGGCCACGT R: CCAACAGAGGACTCTTGGTCT	37.0 °C, 35 cycles	77 bp + 22 bp – wild type homozygote (5G/5G) 98 bp + 77 bp + 22 bp – heterozygote (4G/5G) 98 bp – mutant homozygote (4G/4G)	*Bsll (BseLI)*

The RFLP-PCR analysis of the T-1702A polymorphism of *MMP9* (rs2297864) was performed as follows: First, DNA amplification with 2 μl of 10x PCR buffer (with 15 mM MgCl_2_), 5 μl of 10 mM dNTPs, 2 μl of 25 mM MgCl_2_, 100 pmol of primers (Sigma-Aldrich, St. Louis, Missouri, United States), 1 U of Taq polymerase and 2 μl of genomic DNA. The solution was filled up to 20 μl with double deionized water. The reaction was performed using the Biometra UNO-Thermoblock (Biometra, Goettingen, Germany) thermal cycler according to the procedure: 4 min in 95 °C, followed by 35 cycles of three steps (95 °C for 30 s, 60 °C for 30 s and 72 °C for 30 s) and finally 10 min of elongation in 72 °C. The PCR product was detected in 7% polyacrylamide gel, stained with ethidium bromide and viewed under UV_254nm_ light using the Image Master® VDS from Pharmacia Biotech. Secondly, the PCR product was digested using FastDigest BsaBI (BseJI) (Thermo Fisher Scientific, Waltham, Massachusetts, United States) reacting for 2 h in 65 °C in Biometra's UNO-Thermoblock. The *Bsa*BI *(BseJI)* restriction enzyme digestion products were separated through electrophoresis in 7% polyacrylamide gel, stained with ethidium bromide and viewed under UV_254nm_ light using the Image Master® VDS.

The RFLP-PCR analysis of the *-*675 polymorphism of *SERPINE1* (rs1799889) was performed as follows: First, DNA amplification with 2 μl of 10x PCR buffer (with 15 mM MgCl_2_), 0.5 μl of 10 mM dNTPs, 2 μl of 25 mM MgCl_2_, 50 pmol of primers (from Sigma-Aldrich), 1 U of Taq polymerase and 3 μl of genomic DNA. The solution was filled up to 20 μl with double deionized water. The reaction was performed using the Biometra UNO-Thermoblock thermal cycler according to the procedure: 4 min in 95 °C, followed by 35 cycles of three steps (95 °C for 30 s, 62 °C for 30 s and 72 °C for 30 s) and finally 10 min of elongation in 72 °C. The PCR product was detected in 7% polyacrylamide gel, stained with ethidium bromide and viewed under UV_254nm_ light using the Image Master® VDS from Pharmacia Biotech. Secondly, the PCR product was digested using FastDigest BsaBI (BseJI) from Thermo Fisher Scientific reacting for 2 h at 37 °C in Biometra's UNO-Thermoblock. The *Bsa*BI *(BseJI)* restriction enzyme digestion products were separated through electrophoresis in 7% polyacrylamide gel, stained with ethidium bromide and viewed under UV_254nm_ light using the Image Master® VDS.

In order to assess the accuracy of performed analysis, 10% of samples were chosen randomly for repeated assay.

### 2.5 Statistical analysis

Correlation between analyzed polymorphisms and gene expression has been assessed using Kruskal–Wallis analysis. The correlations between the analyzed polymorphisms and clinical parameters was evaluated using χ^2^ distribution, as well as Kruskal–Wallis analysis and Spearman's rank correlation coefficient. Statistical calculations were performed using STATISTICA 10 software from StatSoft and IBM SPSS 19 (StatSoft, Inc., Tulsa, OK, USA).

## 3 Results

### 3.1 Correlation between polymorphism distribution and gene expression

No correlation between the genotype distribution of T-1702A polymorphism and expression of *MMP9* (*p* = 0.360) was observed. Using the Kruskal-Wallis test has not proven any correlation between the −675 polymorphism and *SERPINE1* expression either. Genotype distribution for each polymorphism is presented in Table 2, showing results that do not deviate from values expected from literature.

**Table 2:** Genotype distribution of analyzed polymorphisms in a group of 35 randomly selected EC patients, presented as percentages. TT - homozygous wild-type allele, AA - homozygous mutated allele, TA – heterozygous allele, 5G/5G - homozygous wild-type allele; 4G/4G - homozygous mutated allele; 4G/5G - heterozygous allele.

Polymorphism	Genotype	Number of patients	%
**polymorphism of *MMP9* T1702A** **rs2297864**	TT	10	28.6
	AA	8	22.9
	TA	17	48.6
**polymorphism of *SERPINE1* -675 4G/5G** **rs1799889**	5G/5G	6	17.1
	4G/4G	10	28.6
	4G/5G	19	54.3

### 2.2 Correlation between polymorphisms and clinical-pathological factors

A positive correlation between the expression of −675 polymorphic form of *SERPINE1* and alcohol abuse has been found, with a *p*-value of 0.026. Additionally, a correlation between the −675 polymorphism and the subtype of EC developed by the patient has been shown (*p* = 0.001). We have observed an interdependence between the expression levels of −675 and T-1702A polymorphisms (*p* = 0.048). The above results were achieved using χ^2^ distribution, as well as Kruskal-Wallis.

By using Spearman's rank correlation coefficient analysis, a positive correlation between the T1702A polymorphism of *MMP9* and alcohol consumption has been shown, with a *p-*value below 0.022. A correlation between *MMP9* and miRNA 134 gene has been shown in our previous research (*p* = 0.037). All patient information, including age, AJCC system, alcohol consumption, gender, lymph node metastasis, family history of cancer, packs of cigarettes smoked per day, other cancers, smoking status and years of smoking is presented in Table 3.

**Table 3:** Clinical and pathological characteristics for patients (n = 35) with EC.

Characteristics		Patients, n (%)	P-value for T1702A *MMP9* (rs2297864)	P-value for −675 *SERPINE1* (rs1799889)
Gender	female	12 (34.3)	P = 0.706	P = 0.464
	male	23 (65.7)		
Age, year^a^	<45	3 (8.6)	P = 0.288	P = 0.370
	45–55	7 (20)		
	56–65	16 (45.7)		
	>65	9 (25.7)		
Type of EC	SCC	16 (45.7)	P = 0.127	P = 0.0008†
	AC	17 (48.6)		
	BSCC	1 (2.9)		
	CS	1 (2.9)		
AJCC system	I + II	13 (37.1)	P = 0.269	P = 0.297
	III + IV	17 (48.6)		
	No data	5 (14.3)		
Lymph node metastasis	Negative	21 (60)	P = 0.708	P = 0.804
	Positive	9 (25.7)		
	No data	5 (20.6)		
Family history of cancer	Yes	3 (8.6)	P = 0.062	P = 0.252
	No	32 (91.4)		
Other cancers	Yes	9 (25.7)	P = 0.316	P = 0.713
	No	26 (74.3)		
Alcohol consumption	Rarely	21 (60)	P = 0.581	P = 0.026†
	Often	14 (40)		
Smoking status	Never	10 (28.6)	P = 0.826	P = 0.478
	Ever	25 (71.4)		
Number of pack of cigarettes/day	0	10 (28.6)	P = 0.586	P = 0.375
	≤2	19 (54.3)		
	>2	6 (17.1)		
Years of smoking	<20	6 (17.1)	P = 0.467	P = 0.210
	21–30	7 (20)		
	>31	11 (31.4)		

a Range, 30–78 years. P < 0.05. EC, esophageal cancer; AJCC, American Joint Committee on Cancer; SCC, squamous cell carcinoma; BSCC, basaloid SCC; ASC, adenosquamous carcinoma; CS, chondrosarcoma.

## 4 Discussion

The following study is a continuation of our previous research, the goal of which was to select genes potentially correlated with etiopathogenic factors for esophageal carcinoma (EC) in polish patients, classified as part of the Caucasian population. As part of the project, genes associated with the metastatic process: *MET*, *MMP9*, *PDGFA*, *SERPINE1* and miRNA genes: miR-21, miR-134, miR-205 and miR-495 were selected for analysis. miR-495 did not show expression in any of the samples. During this research, *MMP9*, *SERPINE1* and miR-134 expression were discovered to be correlated with pathological factors for EC. According to the literature, such correlation has been observed for *MMP9* and mir-134 for the first time [19].

In order to confirm our earlier results for etiopathogenesis-related genes in EC, we decided to perform polymorphic allele analysis for *SERPINE1* and *MMP9.* Specifically, the T1702A polymorphism (rs2297864) was chosen for *MMP9* and *-*675 4G/5G (rs1799889) for *SERPINE1.*

The −675 polymorphism has shown correlation between its expression and alcohol abuse, as well as EC histopathological subtype. Expression of the T1702A polymorphism was found to be associated with alcohol consumption. The two analyzed polymorphic alleles have also shown correlation between each other.

The Kruskal–Wallis test has not shown any correlation between genotype distribution for the T-1702A polymorphism and *MMP9* expression or the −675 polymorphism and *SERPINE1* expression.

A relationship between the analyzed polymorphic allele forms has been found. Spearman's rank correlation coefficient has shown a positive correlation between T1702A expression and alcohol consumption. The correlation between *MMP9* and miR-134 genes described in the previous article has been confirmed.

It has been discovered that *-*675 (rs1799889) plays a role in genetic thrombosis and portal hypertension. It has been proposed that it may increase inflammation response, which is considered the biomolecular base for these diseases [22]. Severe deficiency of plasma *SERPINE1* can lead to recurrent bleeding episodes. There are also noted cases of intermediate deficiency of *SERPINE1* (in this case caused by heterozygous *SERPINE1* locus 4G, 5G deletion and insertion allele) leading to similar clinical outcomes [23]. Recent evidence leads to the conclusion that the 4G/4G variant of *-*675 (rs1799889) 4G/5G polymorphism may be a predictive factor for unfavorable chemotherapy response in testicular cancer (TC) patients. It has been suggested that this may lead to a fine-tuning of the International Germ Cell Consensus Classification prognosis for TC [24]. No association has been found between any of the *SERPINE1* polymorphisms and the onset of ovarian cancer. It has however been demonstrated that the *SERPINE1* -675 4G/4G polymorphism may play a role in endometrial carcinomas, while the *-*675 (rs1799889) polymorphism is associated with a higher risk of various other types of endometrial cancer [25,26]. In the case of breast cancer (BC), *SERPINE1* levels have been associated with overall patient survival rates, without any correlation with the incidence of the disease or its clinical outcome [27]. Combined meta-analysis of data from Caucasians and East Asians has proven that the *-*675 (rs1799889) A/G polymorphism may be a predisposing factor for venous thromboembolism [28]. In hepatocellular carcinoma, elevated plasma levels of SERPINE1 in patients with the 4G/4G polymorphism was found to be associated with reduced survival after transcatheter arterial chemoembolization, compared to patients with 5G/5G or 4G/5G genotype [16]. Meta-analysis conducted on 99 studies involving 62 739 cases in the Caucasian and Asian populations has shown that various polymorphisms of *SERPINE1* may be used as biomarkers for atherosclerotic diseases, including atherosclerosis, coronary artery disease, myocardial infarction (MI) and cerebral infarction. However, a study done on the German population has shown marginal or no relationship between different *SERPINE1* haplotypes and MI [29,30]. Another meta-analytical study has shown that among women, the *SERPINE1* 2675 4G/5G polymorphism plays a likely role in the pathogenesis of pulmonary embolism. *SERPINE1* -675 (rs1799889) polymorphism has also been associated with susceptibility to chronic obstructive pulmonary disease, however, research conducted on the Chinese Han population did not show such association [29,31,32]. The *-*675 (rs1799889) polymorphism has been associated with higher a risk of Avascular necrosis (AVN, osteonecrosis) of the femoral head [33]. As one study suggests, mothers carrying the *SERPINE1* (−675 4G/5G) polymorphism have a higher risk of developing preeclampsia [34]. In a Turkish study, it has been shown that the *-*675 (rs1799889) polymorphism is associated with the development of lung cancer in the local population [30]. It has been suggested that *SERPINE1* can be a prognostic factor for BC patients, as shown by a study performed on the Chinese population [35].

There aren't many reports available on the *MMP9* T-1702A (rs2297864) polymorphism. No correlation between susceptibility to asthma or airway remodeling was found for the *MMP9* T-1702A. polymorphism [36,37]. Introduction of a homozygote for the aforementioned polymorphism can be a predisposing factor in sarcoidosis [38]. The *MMP9* T-1702A polymorphism plays a role in the development of depression [39]. Bobińska et al. have confirmed the aforementioned research. Researchers have observed an increased expression among persons with diagnosed clinical depression, compared to the control group. The authors suggest that the level of expression of *MMPs* (including *MMP9*) could be used as markers for depression and somatic diseases [40]. Manifestation of the TT homozygote genotype of *MMP9* is associated with a significant increase in the probability of age-related macular degradation (AMD) but does no effect on the progression of the illness [41].

## 5 Conclusions

The findings made during this study suggest that there exists a possible association between the occurrence of the *SERPINE1* -675 4G/5G (rs1799889) polymorphism and the increased risk of EC in the Caucasian population. To confirm the results presented in this paper, additional research will be required. That includes analysis of a larger sample pool of EC patients. Analyzing data from different populations from around the world is also recommended. To achieve a broader understanding of the role of allele polymorphisms of *SERPINE1* and *MMP9* in EC, it is recommended to analyze additional polymorphisms of these genes.

## Author contributions

J.S. and A.A.K–B. conceived and designed the experiments; A.A.K–B and K.H. performed the experiments; A.A.K–B. analyzed the data; A.A.K–B. write original the manuscript, A.A.K–B.; J.B. and J.S reviewed the manuscript. All authors have read and agreed to the published version of the manuscript.

## Funding

This research was funded by 10.13039/100009590Medical University of Lodz, Poland, Faculty of Health Sciences, grant number 502–03/6-099-01/502-64-089, 503/6-086-01/503-61-001 and 503/6-099-01/503-61-001-19-00.

## Institutional review board statement

Not applicable.

## Informed consent statement

Informed consent was obtained from all subjects involved in the study. Written informed consent has been obtained from the patients to publish this paper.

## Data availability statement

The data presented in this study are available on request from the corresponding author.

## Declaration of competing interest

The authors declare no conflict of interest.
